# Towards the Prediction of Responses to Cancer Immunotherapy: A Multi-Omics Review

**DOI:** 10.3390/life15020283

**Published:** 2025-02-12

**Authors:** Weichu Tao, Qian Sun, Bingxiang Xu, Ru Wang

**Affiliations:** 1 School of Exercise and Health, Shanghai University of Sport, Shanghai 200438, China; tao.weichu@outlook.com (W.T.); sunq0423@163.com (Q.S.); 2 Key Laboratory of Hebei Province for Molecular Biophysics, Institute of Biophysics, School of Health Science & Biomedical Engineering, Hebei University of Technology, Tianjin 300130, China

**Keywords:** tumor immunotherapy, multi-omics, machine learning, biomarker

## Abstract

Tumor treatment has undergone revolutionary changes with the development of immunotherapy, especially immune checkpoint inhibitors. Because not all patients respond positively to immune therapeutic agents, and severe immune-related adverse events (irAEs) are frequently observed, the development of the biomarkers evaluating the response of a patient is key for the application of immunotherapy in a wider range. Recently, various multi-omics features measured by high-throughput technologies, such as tumor mutation burden (TMB), gene expression profiles, and DNA methylation profiles, have been proved to be sensitive and accurate predictors of the response to immunotherapy. A large number of predictive models based on these features, utilizing traditional machine learning or deep learning frameworks, have also been proposed. In this review, we aim to cover recent advances in predicting tumor immunotherapy response using multi-omics features. These include new measurements, research cohorts, data sources, and predictive models. Key findings emphasize the importance of TMB, neoantigens, MSI, and mutational signatures in predicting ICI responses. The integration of bulk and single-cell RNA sequencing has enhanced our understanding of the tumor immune microenvironment and enabled the identification of predictive biomarkers like PD-L1 and IFN-γ signatures. Public datasets and machine learning models have also improved predictive tools. However, challenges remain, such as the need for large and diverse clinical datasets, standardization of multi-omics data, and model interpretability. Future research will require collaboration among researchers, clinicians, and data scientists to address these issues and enhance cancer immunotherapy precision.

## 1. Introduction

Cancer immunotherapy has emerged as one of the most promising treatment modalities in the fight against malignancies, harnessing the power of the immune system to target and eliminate tumor cells. Immune checkpoint inhibitors (ICIs), which block immune checkpoint proteins that restrict immune responses, have shown success in treating various cancers, including melanoma, non-small-cell lung cancer (NSCLC), and renal cell carcinoma. Despite the transformative potential of ICIs, the clinical response to immunotherapy remains highly variable, with a significant proportion of patients experiencing limited or no benefit from treatment. This heterogeneity in treatment outcomes underscores the urgent need for predictive biomarkers that can identify patients most likely to respond to immunotherapy.

**Figure 1:**
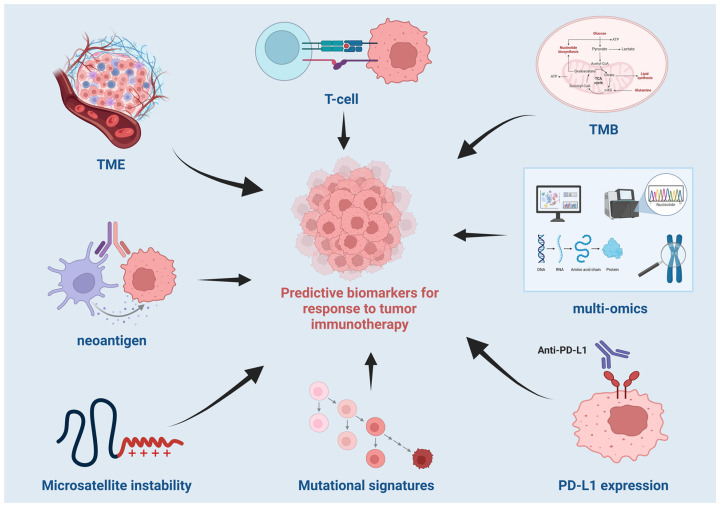
Decoding tumor immunotherapy: a multi-dimensional perspective on key biomarkers and treatment response.

Over the past decade, the application of multi-omics approaches has revolutionized our understanding of tumor biology and its complex interactions with the immune system. Multi-omics, which integrates data from genomics, transcriptomics, proteomics, and epigenomics, provides a comprehensive view of the molecular landscape of tumors and their immune microenvironment. This integrated approach has the potential to uncover novel biomarkers and mechanisms that predict response to immunotherapy, offering personalized treatment strategies tailored to individual patients. Genomic alterations, such as tumor mutation burden (TMB), microsatellite instability (MSI), and immune checkpoint gene expression, have emerged as key factors influencing the efficacy of ICIs. Moreover, the combination of multi-omics data with clinical features allows for the development of sophisticated predictive models that could guide clinical decision-making and improve patient outcomes. The predicted relationship is shown in Figure 1.

This review aims to explore the potential of multi-omics data in predicting responses to cancer immunotherapy. We will discuss the various layers of omics data, including genomic, transcriptomic, proteomic, and epigenomic information, and how their integration can offer insights into the immune mechanisms underlying tumor progression and treatment resistance. Additionally, we will examine the role of machine learning (ML) and deep learning techniques in developing predictive models, highlighting their ability to analyze complex, high-dimensional data and provide more accurate predictions of immunotherapy outcomes. By reviewing the current advancements in the field, this paper seeks to outline the promising future of multi-omics-based prediction models in cancer immunotherapy, offering new avenues for precision oncology.

## 2. Results

### 2.1. Predictive Biomarkers for Response to Tumor Immunotherapy

#### 2.1.1. Sequence-Based Biomarkers

Sequence-based biomarkers, primarily derived from genomic data, have emerged as crucial tools in predicting responses to immune checkpoint inhibitor therapy [1,2]. TMB, neoantigen, MSI, and mutational signatures are among the most widely studied sequence-based biomarkers [3,4]. These biomarkers provide valuable insights into the genetic landscape of tumors and their potential to elicit immune responses, thus guiding personalized treatment strategies. However, while these biomarkers have shown promise, their predictive value remains limited when applied individually, prompting the exploration of more complex, multi-dimensional models that integrate genomic features with other omics data to enhance predictive accuracy (All the medical terms of full name and explanation have shown in the Supplementary Table S1).

##### Tumor Mutation Burden

TMB, a measure of the total number of mutations within a tumor genome, has emerged as a promising predictive biomarker for ICI response in cancer immunotherapy. TMB correlates with immune system activation and the likelihood of successful ICI therapy. Studies, including McLean et al. (2024), Meng et al. (2024), and Rodrigo et al. (2024), have shown that high TMB is associated with better clinical outcomes, such as improved overall survival (OS), progression-free survival (PFS), and objective response rate (ORR), in cancers like NSCLC, Merkel cell carcinoma (MCC), CRC, and breast cancer [5,6,7].

However, the relationship between TMB and immunotherapy response is complex. While high TMB is generally a reliable indicator of ICI efficacy, its predictive value varies across tumor types, patient populations, and treatment regimens. For example, high TMB correlates with better responses to ICIs in NSCLC and melanoma, where it increases the likelihood of neoantigen generation that the immune system can target. However, in gastric cancer and pancreatic cancer, the predictive value of TMB is less clear. Garon et al. (2015) found pembrolizumab to be effective in NSCLC patients with high TMB, but Marabelle et al. (2020) showed that TMB was less predictive in gastric cancer [8,9]. In particular, for Merkel cell carcinoma, immune checkpoint inhibitors, especially PD-1 inhibitors, are the first-line systemic therapy. High TMB (TMB-H) has been associated with improved responses to PD-1 inhibitors, whereas the efficacy of PD-L1 inhibitors may vary depending on TMB status [10,11]. The differential response to PD-1 and PD-L1 inhibitors in TMB-H and TMB-L tumors may be due to the distinct mechanisms of immune evasion and tumor microenvironment modulation.

The role of TMB as a biomarker has also been explored in rare cancers such as MCC, penile squamous cell carcinoma (PSCC), and gallbladder cancer (GBC). Although studies on these cancers are less conclusive, they suggest that TMB could still provide valuable predictive insights. A recent study found that high PD-L1 expression in carcinomas lacking the SWI/SNF-related matrix-associated tumor suppressor gene which acts as an independent regulator of chromatin subfamily B member 1 (also known as INI-1 or BAF47, located on chromosome 22q11.2) was associated with favorable immune checkpoint inhibitor response, even in cases with low TMB. Mutations or loss of SMARCB1 can lead to uncontrolled cell growth and are associated with various cancers. This provides valuable predictive insights into the complexity of tumor microenvironments [12].

TMB’s predictive value can also vary by ethnicity. A study by Sisoudiya SD et al. (2024) suggests that the effectiveness of TMB combined with PD-L1 expression may differ across populations [13]. These findings underline the importance of considering genetic and environmental factors in personalized immunotherapy approaches. Despite evidence supporting TMB, some studies, like Li et al. (2022), show that high TMB does not always correlate with favorable outcomes, as seen in some CRC cases [14]. This suggests that additional genetic and molecular factors must be considered when using TMB to predict immunotherapy efficacy.

Combining TMB with other biomarkers, such as PD-L1 expression, has been shown to improve prediction accuracy. In NSCLC, high TMB and PD-L1 expression are often associated with better responses to ICIs. Ozdogan et al. (2024) and Nguyen et al. (2024) highlight this synergy, providing a more nuanced prediction. Combining TMB with MSI and deficient mismatch repair (dMMR) further enhances predictive power, especially in cancers like colorectal and GBC [15,16].

Meta-analyses by McLean et al. (2024) and Nguyen et al. (2024) support the use of TMB combined with other immune-related biomarkers like PD-L1 and MSI for ICI response prediction [5,15]. These studies emphasize the value of multi-biomarker approaches. However, challenges remain in clinical applications due to a lack of standardized thresholds for defining high TMB across cancer types. Some studies use thresholds like 10 or 20 mutations per megabase, but this variability complicates clinical interpretation.

In conclusion, TMB is a promising biomarker for predicting immunotherapy response, particularly in cancers like NSCLC, melanoma, and CRC. Its predictive value is enhanced when combined with other biomarkers like PD-L1 and MSI. However, its utility varies across cancer types and ethnic populations, emphasizing the need for personalized approaches to immunotherapy.

##### Neoantigen

Neoantigens are effective predictors of ICI efficacy, helping identify patients likely to respond to treatment. Among the various biomarkers, neoantigens have emerged as promising sequence-based predictors of ICI response. Neoantigens, which are novel peptide sequences resulting from tumor-specific mutations, play a crucial role in eliciting robust antitumor immune responses. Here, we delve into the significance of neoantigens as predictive biomarkers for ICI therapy, exploring their identification, prediction algorithms, clinical applications, and the challenges associated with their use.

Neoantigens are tumor-specific antigens that arise from non-synonymous somatic mutations in cancer cells. Unlike tumor-associated antigens, neoantigens are not present in normal tissues, making them ideal targets for immune recognition without the risk of autoimmunity. However, while the presence of neoantigens is predictive of immune checkpoint inhibitor (ICI) response, as seen in cancers like desmoplastic melanoma, Merkel cell carcinoma (MCC), and cutaneous squamous cell carcinoma (CSCSC), the antitumoral effect is independent of autoimmune toxicity. Autoimmune toxicity is thought to be elicited by the re-activation of pre-existing autoimmune cytotoxic T lymphocyte (CTL) clones in patients, rather than the antitumoral immune response itself. Chen et al. (2021) emphasize the pivotal role of neoantigens in cancer immunotherapy, highlighting advancements in sequencing technologies and neoantigen prediction algorithms that have enhanced the identification and prioritization of these targets [17]. The ability to tailor immunotherapies based on individual neoantigen profiles holds the promise of personalized cancer treatment, potentially increasing the efficacy and reducing the toxicity of ICIs.

The prediction of neoantigens relies heavily on next-generation sequencing (NGS) technologies, such as whole-genome sequencing (WGS), whole-exome sequencing (WES), or targeted gene panels, along with sophisticated bioinformatics tools. De Mattos-Arruda et al. (2020) provide a systematic review of neoantigen prediction frameworks, discussing the evolution of computational pipelines that integrate multiple features beyond mere peptide–MHC binding affinity. Modern approaches incorporate variant allele fraction, gene expression levels, and the clonality of mutations to enhance prediction accuracy [18]. Chen et al. (2021) outline the two primary strategies for neoantigen prediction: direct prediction of peptide ligands eluted from peptide–MHC complexes and the combination of NGS with prediction algorithms [17]. These methodologies, applied through WGS, WES, or targeted panels, have significantly improved the ability to detect and prioritize neoantigens, facilitating the design of effective cancer vaccines and adoptive T-cell therapies.

A high neoantigen load (NAL) has been associated with increased T-cell infiltration and enhanced antitumor immunity. Mao et al. (2023) demonstrates that cancers with a high response to ICIs typically exhibit greater T-cell infiltration and higher NAL, suggesting that neoantigen burden is a critical determinant of immunotherapy success [19]. Furthermore, Jiang et al. (2020) introduced the immunotherapy score (ITS), a composite biomarker that integrates NAL with TMB and other genetic factors [20]. The ITS model outperforms traditional TMB in predicting responses to ICIs, underscoring the importance of neoantigens in enhancing predictive accuracy.

Specific genetic mutations contribute to the generation of highly immunogenic neoantigens. Studies by Petrelli et al. (2020) and Mao et al. (2023) identify mutations in genes such as USH2A, ZFHX4, and PLCO as being highly correlated with enhanced tumor immunogenicity and improved outcomes with ICIs [19,21]. These mutations facilitate the formation of neoantigens that are more likely to be recognized by the immune system, thereby strengthening the antitumor response. Additionally, Litchfield et al. (2021) highlight that clonal TMB, which reflects the number of neoantigens derived from clonal mutations, is the strongest predictor of checkpoint inhibitor response, further emphasizing the role of specific genetic alterations in immunotherapy efficacy [22].

Personalized neoantigen vaccines represent a cutting-edge approach in cancer immunotherapy, aiming to induce neoantigen-specific immune responses. Khan et al. (2021) reviewed clinical trials involving neoantigen-based vaccines for glioblastoma (GBM), demonstrating high immunogenicity and safety profiles. Their review also includes data from trials involving a total of 24 GBM patients, highlighting the promising potential of these vaccines in this aggressive cancer type [23]. These personalized vaccines, when combined with standard treatments, show promise in improving clinical outcomes for GBM patients. The success of these vaccines in inducing robust T-cell responses highlights the potential of neoantigen-based strategies in personalized medicine, offering a tailored approach to cancer treatment that targets the unique mutational landscape of each tumor. While the majority of studies have focused on GBM, there is growing interest in exploring the application of neoantigen vaccines in other solid tumors, including melanoma, CRC, and gastric cancer [24,25,26].

##### Microsatellite Instability

MSI is an important molecular biomarker in cancer. MSI results from defects in the MMR system, causing an accumulation of mutations that increase the tumor’s visibility to the immune system and enhance the potential for immunotherapy responses. MSI-high (MSI-H) tumors generally show better survival outcomes and are more responsive to ICIs compared to microsatellite-stable (MSS) tumors.

Studies consistently highlight MSI as a crucial factor in guiding immunotherapy. Pietrantonio et al. (2019) conducted a meta-analysis across multiple gastric cancer trials, showing that MSI-H tumors have improved survival with ICIs, suggesting a preference for immunotherapy over chemotherapy [26]. Yoon et al. (2022) corroborated these findings, emphasizing MSI status as a predictive biomarker and proposing that combining MSI with PD-L1 expression assessment could refine treatment strategies in gastrointestinal cancers [27]. Notably, a negative prognostic impact of male sex on overall survival (OS) has also been documented in stage II colorectal cancer patients with dMMR/MSI-H tumors.

In endometrial cancer, Bogani et al. (2024) explored the improved progression-free survival (PFS) of MSI-H tumors when treated with combined chemotherapy and immunotherapy, which is less commonly studied compared to monotherapy with immune checkpoint inhibitors (ICIs) [28]. This highlights the potential for a more personalized treatment strategy in endometrial cancer. Additionally, the prognostic negative impact of male sex on overall survival (OS) has also been reported in dMMR/MSI-H colorectal cancer (CRC) at stage II [29].

Similarly, MSI status is predictive of better outcomes in CRC. Zhou et al. (2023) studied MSI-H CRC patients treated with neoadjuvant immunotherapy, demonstrating favorable responses even before metastasis [30]. This finding complements previous research by emphasizing MSI’s role in guiding therapy early in the treatment process.

In ovarian cancer, Mitric et al. (2023) recommended integrating MSI and MMR deficiency testing into clinical practice, showing that MSI-H tumors may also respond well to immunotherapy, despite ovarian cancer’s typically limited focus on MSI [31]. This underscores the growing importance of MSI testing across various cancer types.

Formica et al. (2022) examined MSI in CRC, highlighting how MSI status can interact with mutations like KRAS and BRAF, influencing prognosis and treatment response [32]. This suggests the need for a more comprehensive molecular profile to guide CRC treatment.

Gender-specific differences in MSI-H gastric cancer prognosis were explored by Quaas et al. (2022), revealing that male patients had worse survival outcomes than females [33]. This finding adds complexity to MSI research, proposing gender as a factor in MSI-related prognosis.

Lastly, O’Connell et al. (2020) investigated MSI in rectal cancer’s response to chemoradiotherapy (CRT) and found no significant impact, contrasting with findings in immunotherapy [34]. This highlights the distinction between therapeutic modalities and the need for different treatment strategies for MSI-H tumors in various cancer types.

##### Mutational Signatures

Mutational signatures have emerged as pivotal biomarkers with significant potential for guiding personalized treatment strategies. These signatures, characterized by specific patterns of somatic mutations within tumor genomes, provide insights into the mutagenic processes that drive cancer development and can serve as predictive indicators for therapeutic outcomes [35].

Recent advancements in whole-genome sequencing (WGS) have enabled the identification and characterization of numerous mutational signatures across a wide range of cancer types. In a comprehensive study by Alexandrov et al. (2020), 49 single-base-substitution and several other types of mutational signatures were identified across 4645 whole-genome sequences from the Pan-Cancer Analysis of Whole Genomes (PCAWG) Consortium [36]. These signatures are associated with different mutational processes, including DNA repair defects and external exposures like UV radiation or tobacco smoke [36]. By examining these patterns, researchers can better understand the mutational processes that contribute to cancer development, and how these processes might influence the immune system’s ability to recognize and target tumors.

The role of mutational signatures in cancer immunotherapy lies in their potential to predict tumor responsiveness to immune-based treatments, such as ICIs. For instance, tumors exhibiting signatures associated with defective DNA repair mechanisms, such as those involving homologous recombination deficiency (HRD), are often more sensitive to therapies like PARP inhibitors. This is because these tumors accumulate more genetic instability, creating a higher mutational burden that can lead to the generation of neoantigens. Tumors with a high mutational load are thus more likely to respond to ICIs, which work by enhancing the immune system’s ability to detect and destroy cancer cells [37].

Furthermore, mutational signatures associated with APOBEC (Apolipoprotein B mRNA Editing Enzyme Catalytic Subunit) activity have been shown to influence the tumor’s immune microenvironment and its response to immune therapies. APOBEC-induced mutations generate specific mutational patterns, which can result in the production of neoepitopes that may be targeted by T cells [36]. Identifying these signatures helps to predict which cancers are more likely to respond to immune checkpoint blockade therapies, such as anti-PD-1/PD-L1 antibodies.

Another significant application of mutational signature analysis in immunotherapy is in the design of personalized cancer vaccines. Tumors have unique mutational landscapes, and every patient’s cancer genome harbors a distinct set of mutations. By sequencing the mutations within a tumor and identifying the corresponding mutational signatures, researchers can develop personalized vaccines that target tumor-specific neoepitopes, thus enhancing the immune system’s ability to recognize and attack cancer cells [38]. Clinical trials exploring personalized cancer vaccines have shown promising results, with certain tumors exhibiting strong immune responses following vaccination based on mutational signatures.

In conclusion, mutational signature analysis offers valuable insights into the molecular mechanisms driving cancer and provides a powerful tool for predicting responses to cancer immunotherapy. By identifying the specific mutational processes and associated signatures in a patient’s tumor, clinicians can better predict the efficacy of immune-based therapies, leading to more personalized and effective treatment strategies.

#### 2.1.2. Biomarkers Based on Gene Expression Profiles

Gene expression profiling, particularly bulk RNA sequencing and single-cell RNA sequencing technologies, has become a key tool in cancer research for identifying biomarkers and understanding the tumor immune microenvironment. In the field of tumor immunology, bulk RNA sequencing has been extensively applied to identify new biomarkers, among which the expression of PD-L1 is the most studied gene expression biomarker. High PD-L1 expression correlates with better outcomes in patients treated with PD-1/PD-L1 inhibitors, making it an important predictive marker for immune checkpoint therapy [39]. Additionally, the upregulation of interferon-gamma (IFN-γ) response signatures indicates an inflammatory tumor microenvironment (TME), which is more likely to respond to immunotherapy [40]. Conversely, markers of T-cell exhaustion, such as the expression of inhibitory receptors PD-L1 and CTLA-4, can help identify patients who cannot benefit from standard immunotherapy due to immune suppression within the tumor [41]. Studies have shown that tumors expressing high levels of immune-related genes, especially those involving antigen presentation (e.g., MHC Class I and II molecules) and interferon (IFN) signaling pathways, tend to have a better response to ICIs [42].

Single-cell RNA sequencing (scRNA-seq) allows us to describe gene expression at the resolution of individual cells. This technology helps reveal complex interactions between tumor cells and their microenvironment, particularly immune cells involved in regulating tumor growth and immune escape [43]. A key advantage of scRNA-seq is its ability to identify rare cell populations that may play a critical role in tumor progression and resistance to treatment. For example, studies have used scRNA-seq to reveal the diversity of T cells in the TME, distinguishing exhausted, active, or memory T-cell subtypes [44]. Elham Azizi and colleagues also explored the main features of cellular targets for immunotherapy in human breast cancer by generating a deep transcriptional map of immune cell states in human breast tumors using scRNA-seq technology [45]. Other studies have shown that the expression of immune checkpoint molecules such as PD-1, PD-L1, and CTLA-4 can vary significantly across different cell populations within the tumor, highlighting the complexity of immune regulation in the TME [46].

In summary, the integration of bulk RNA sequencing and single-cell RNA sequencing technologies has significantly advanced our understanding of the tumor immune microenvironment, enabling the prediction of predictive biomarkers such as PD-L1 and IFN-γ response signatures, as well as the characterization of immune cell heterogeneity and exhaustion markers like PD-L1 and CTLA-4. These insights are crucial for optimizing immunotherapy strategies and developing personalized cancer treatments.

#### 2.1.3. Biomarkers Based on Epigenomic Profiles

Biomarkers derived from epigenomic profiles are gaining increasing significance in cancer diagnosis, prognosis, and monitoring treatment responses [47]. Abnormal DNA and histone modifications that silence tumor suppressor genes or activate oncogenes have been identified across various cancer models. Analyzing these epigenetic modifications helps identify patterns associated with specific cancer types or subtypes, fostering the development of new biomarkers [48]. High-throughput technologies identifying epigenetic variations enable precise localization and quantification of epigenetic changes and are critical in identifying disease-associated biomarkers.

##### DNA Methylation Profiles

DNA methylation plays a crucial role in tumorigenesis and predicting immunotherapy responses by regulating the TME and gene expression. In cancer, tumor environment-induced methylation undergoes specific changes that can lead to the silencing of tumor suppressor genes and activation of oncogenes [49], ultimately affecting various biological pathways. 5 mC (5-Methylcytosine), a form of DNA methylation typically enriched in gene promoter regions, plays a critical role in regulating gene expression. This focal gain of 5 mC in gene promoters was initially proposed as a non-hereditary means of inactivating tumor suppressor genes. On the other hand, global loss of 5mC is also believed to promote genomic instability [50]. Although significant progress has been made in cancer detection based on methylation over the past few years, most existing markers are still targeted at a single cancer type, and even the most mature markers show diagnostic deficiencies at different tumor stages. Ibrahim, J and colleagues studied the whole-genome methylation profiles of 14 different cancer types using The Cancer Genome Atlas (TCGA) and developed a three-step computational method to select candidate biomarker CpG sites to accurately differentiate tumor types [51]. This provided a comprehensive reference for whole-genome methylation patterns of several of the most common cancer types. Furthermore, some studies have found that the methylation level of certain genes can also predict the response to immunotherapy, such as the methylation status of the PD-L1 gene being related to the efficacy of PD-1/PD-L1 inhibitors. In a published study, a genome-wide analysis was conducted to identify differentially methylated promoters and enhancers associated with response to anti-PD-1 therapy. The research revealed significant differences in methylation levels of pDMRs for CYTIP and TNFSF8 between responders and non-responders, indicating a substantial correlation between methylation levels in these regions and response to anti-PD-1 treatment [52]. In another study, EPIMMUNE Discovery recruited 34 patients with advanced NSCLC who had not received any antitumor treatment [53]. Through whole-genome 850 K methylation chip analysis of tumor tissue DNA, it was found that the methylation levels of 301 CpGs were significantly associated with clinical responses to PD-1 blockade therapy in NSCLC patients. Researchers used these 301 CpG sites as EPIMMUNE epigenomic markers and adopted a supervised classification model of elastic net regularized logistic regression to classify all patients into EPIMMUNE-positive (responsive to treatment) and EPIMMUNE-negative (non-responsive to treatment) groups. Multivariate Cox regression analysis results showed that the EPIMMUNE marker was an independent predictor of PFS and OS in NSCLC patients receiving PD-1 blockade therapy. Additionally, a study by Xu, B et al. discovered that genome-wide DNA methylation signatures play a significant role in predicting responses to cancer immunotherapy [54]. They analyzed DNA methylation features associated with responsiveness and non-responsiveness to ICIs and constructed a support vector machine (SVM) model. This model demonstrated high performance at the pan-cancer level and in specific cancer types, comparable to models based on gene expression, indicating that DNA methylation features can predict ICI treatment responses both at the pan-cancer level and for individual cancer types. These research advances not only deepen our understanding of the role of DNA methylation in the tumor immune microenvironment but also provide a solid foundation for developing new predictive tools and treatment strategies.

##### Histone Modification Profiles

Histone modifications have emerged as crucial regulators in cancer epigenetics. These modifications, including acetylation, methylation, phosphorylation, and ubiquitination, alter chromatin structure and gene transcription activity [55]. For example, histone H3K27me3 is typically associated with gene silencing, while H3K4me3 is linked to gene activation [56]. In cancer, histone modifications play a central role in tumor pathogenesis. One study identified AFAP1-AS1 as a potential biomarker for triple-negative breast cancer (TNBC), associated with cell proliferation and epithelial–mesenchymal transition (EMT) through specific histone modification patterns [57]. In NSCLC, researchers found that SIRT6 expression was inversely correlated with H3K56ac levels, and higher SIRT6 expression was associated with early-stage disease and longer survival [58]. This suggests that analyzing histone modifications can provide valuable insights into cancer progression, with H3K56ac levels serving as potential biomarkers. In liver cancer (HCC), studies found that H3K27me3, H4K20me2, and H4K16ac were linked to poor prognosis, with H3K4me3 levels also indicating worse outcomes in HCC patients [59]. Histone modifications also influence tumor cell immunogenicity and immune escape. For example, inhibiting HDAC6 can activate PD-L1 expression in melanoma [60], and abnormal KMT2A methylation promotes pancreatic cancer development [61]. Furthermore, BRD4, an acetylation reader protein, regulates gene expression in ovarian cancer by recognizing acetylated histones [62]. These studies emphasize the critical role of histone modifications in cancer development and immune regulation, highlighting their potential as biomarkers for cancer diagnosis and treatment.

### 2.2. Cohorts and Datasets for Studying Tumor Immunotherapy Responses

Cohorts and datasets play a pivotal role in advancing our understanding of tumor immunotherapy responses, particularly in the development of predictive biomarkers for ICIs. Large-scale clinical datasets, such as TCGA and the International Cancer Genome Consortium (ICGC), provide valuable genomic and clinical data that facilitate the identification of molecular signatures associated with ICI efficacy. However, these datasets often lack detailed clinical annotations related to ICI treatment, limiting their full potential. Recent efforts have focused on establishing dedicated cohorts of patients undergoing ICI therapy, allowing for more precise correlation between molecular features and therapeutic outcomes. Additionally, multi-omics datasets, integrating genomic, transcriptomic, proteomic, and epigenomic data, are increasingly used to develop robust predictive models. The integration of diverse datasets across various cancer types has also spurred the development of pan-cancer models, enhancing the generalizability and predictive power of immunotherapy response predictions. Despite challenges such as data heterogeneity and cohort size, these resources remain essential for refining personalized treatment approaches. However, clinical studies combining ICI with anti-methylating or anti-acetylating agents have often failed to achieve the expected outcomes. One possible reason for these failures is the complex interplay between epigenetic modifications and immune responses. For example, while certain molecular signatures have been identified as predictive of ICI efficacy, the presence of specific epigenetic modifications may not consistently correlate with treatment response across different cancer types. Additionally, the heterogeneity of tumor microenvironments and the variability in immune cell infiltration can further complicate the effectiveness of combined therapies. Future research should focus on identifying more precise biomarkers and optimizing combination strategies to enhance the efficacy of ICI-based treatments.

#### 2.2.1. Designing Cohorts for Studying the Tumor Immunotherapy Responses

Clinical trials have been a cornerstone of cancer research, playing a vital role in advancing therapeutic strategies and improving prognostic predictions. However, the process of cohort selection in cancer clinical trials is nuanced and varies based on the disease context, the type of intervention, and the specific goals of the study. Clinical trials in cancer research often rely on rigorous selection criteria that incorporate demographic factors, clinical history, genetic makeup, and disease stage to form representative and homogenous patient populations. The diversity of clinical cohorts ensures that findings are generalizable across different subgroups, yet there is increasing recognition of the need for precision in cohort selection to address specific molecular and genetic factors that influence tumor behavior and treatment outcomes.

For instance, in studies focusing on CRC, patient cohorts are typically stratified based on clinical features such as age, sex, family history, and the presence of comorbidities, alongside the stage and molecular characteristics of the disease. The inclusion criteria often involve patients with defined disease stages, commonly those with early or metastatic cancer, depending on the focus of the clinical trial. Such stratification ensures that the trial findings reflect the heterogeneity of the disease and its response to different therapeutic modalities. Moreover, a growing number of clinical studies have incorporated genetic screening into patient selection, as specific mutations or molecular markers are increasingly used to tailor interventions. For example, mutations in KRAS, BRAF, or PIK3CA genes in CRC are often used as biomarkers for selecting patients eligible for targeted therapies, ensuring that the treatment is both relevant and likely to provide benefit to the patient population.

In addition, selection criteria in clinical trials are evolving to address the increasing importance of the TME and immune profiles in predicting patient outcomes and treatment efficacy. Many recent studies now consider ICIs as part of treatment regimens, and the presence of specific immune cells, TMB, and MSI status are used to select patients for immunotherapy. This selection process is crucial, as it allows for the identification of patients who are most likely to benefit from immunotherapy based on their immune status. For example, studies have shown that the presence of tumor-infiltrating lymphocytes (TILs), particularly CD8+ T cells, is associated with better responses to ICIs in various cancers. Additionally, a high level of TILs has been identified as a valuable predictor of response to ICI therapies in clinical trials. However, the specific immune cell profiles and their impact on treatment outcomes can vary significantly across different cancer types and patient cohorts. The work of Gobbini et al. (2024), which showed that interval treatment between immunotherapy sessions, including chemotherapy, targeted therapy, or radiotherapy, may bring survival benefits to patients with advanced non-small-cell lung cancer (NSCLC) who receive immune rechallenge after resistance to first-line immunotherapy. This suggests that the incorporation of diverse treatment modalities and the consideration of treatment history can further refine patient selection and improve outcomes [63].

Furthermore, clinical trials are increasingly utilizing patient-derived xenografts (PDXs) and organoids derived from patient tumors, which are used to mimic the human TME and response to treatment in vitro and in vivo. These models, along with genetically engineered animal models, are essential for understanding tumor biology and for testing new therapies before clinical application. The use of these models in clinical trials, as seen in studies involving ovarian and pancreatic cancers, allows for the creation of more relevant and personalized treatment protocols [64,65,66,67].

However, while clinical trial cohorts are integral for developing new treatments, the challenge lies in ensuring their representativeness and accessibility. Diverse cohorts, especially those that include under-represented groups, are needed to enhance the applicability of trial findings across different populations. This has prompted initiatives to ensure that clinical trials reflect a more global and ethnically diverse patient base, acknowledging the ethnic and genetic variations that influence cancer risk, progression, and treatment response.

The role of multi-omics data in clinical trial cohort development cannot be underestimated. As exemplified in studies such as those by Vasaikar et al. (2018), clinical trials increasingly leverage multi-omics technologies to deepen the understanding of cancer [68]. By integrating genomic, transcriptomic, proteomic, and epigenomic data, these trials aim to identify new molecular targets and refine patient selection based on their unique molecular profiles. These efforts underscore the growing importance of omics-driven clinical trials in predicting individual responses to cancer treatments, allowing for more personalized and targeted interventions.

#### 2.2.2. Application of Public Cancer Data Sources in Developing the Prediction Models for Tumor Immunotherapy Responses

Publicly available cancer data, particularly multi-omics datasets from large-scale initiatives like TCGA, have played a crucial role in identifying key molecular signatures that predict how tumors respond to immunotherapy.

Multi-omics datasets provide valuable insights into the genetic alterations, immune landscape, and molecular pathways that govern tumor response to immunotherapy. For example, genomic data from TCGA, including WGS and targeted sequencing, help identify mutations in genes such as KRAS, TP53, and PIK3CA, which influence tumor progression and immune evasion. These mutations can serve as biomarkers to predict the efficacy of immunotherapy. By combining genomic data with transcriptomic data (such as RNA sequencing), researchers can identify differentially expressed genes that are associated with immune activation or suppression, thereby highlighting potential therapeutic targets to enhance immune responses.

Public cancer datasets, such as TCGA, contain large patient cohorts that facilitate the development of generalized predictive models not limited by small sample sizes. By analyzing TMB, gene expression, and immune infiltration levels, researchers have developed models to predict immunotherapy efficacy. The relationship between TMB and immunotherapy efficacy has been extensively studied. High TMB is associated with increased neoantigen load, enhancing immune recognition and response. Combining TMB with immune checkpoint markers (e.g., PD-L1 expression) further improves the ability to predict clinical responses. By integrating genetic alterations and immune infiltration data, researchers can predict which patients are more likely to benefit from therapies like anti-PD-1 or anti-CTLA-4 agents [69].

Public datasets have also enabled the discovery of immune cell profiles as important predictors of ICI efficacy. Researchers have leveraged tools such as CIBERSORT and TIMER to analyze immune cell infiltration within the TME using publicly available data. These datasets have revealed distinct immune cell signatures associated with better or worse immunotherapy outcomes. For example, high infiltration of CD8+ T cells has been linked to favorable responses, while an abundance of regulatory T cells (Tregs) or myeloid-derived suppressor cells (MDSCs) correlates with immune suppression and poor therapeutic outcomes. By integrating gene expression data from TCGA with immune infiltration profiles, public datasets have facilitated the development of predictive models that identify immune-related biomarkers and propose treatment strategies to modulate the TME, enhancing immunotherapy efficacy.

By integrating multi-omics data, public cancer datasets have also facilitated the identification of novel therapeutic targets. These datasets allow researchers to link genetic mutations with immune cell profiles and clinical outcomes, leading to the discovery of underexplored biomarkers and potential therapeutic targets. For example, CXCL8, CLEC9A, and TAB2 have been identified as crucial mRNA biomarkers related to immune microenvironment alterations in cervical cancer. Such findings highlight the potential of public datasets to inform new therapeutic strategies, including immune modulation and combination therapies, that can help overcome resistance in tumors that do not respond well to monotherapy [70,71,72,73].

As immunotherapy continues to evolve, the integration of public cancer datasets remains crucial for enhancing our understanding of tumor immunology and refining treatment strategies. By leveraging large, multi-omics datasets, public databases enable researchers to develop more accurate, robust prediction models for immunotherapy responses. These models will play a key role in the eventual goal of delivering more personalized and effective cancer treatments. Through continuous exploration of these datasets, the field of cancer immunotherapy will likely experience further breakthroughs, improving outcomes for patients through better-targeted therapeutic approaches.

#### 2.2.3. Published Clinical Cohorts on Cancer Immunotherapy

Previous cohort studies have provided substantial clinical evidence, yet many unresolved issues remain, particularly regarding the long-term effects of tumor treatment and the challenge of treatment resistance. These issues are critical for the field of immunotherapy. To address these challenges and develop more precise immunotherapeutic strategies, it is essential to track larger, longer-term cohorts based on previously published studies. This section will review some of the more important and well-known published clinical cohorts (Table 1).

The CheckMate 057 trial (2015) evaluated Nivolumab versus Docetaxel in patients with advanced non-squamous NSCLC who had previously received platinum-based chemotherapy [69]. With 582 participants, the study demonstrated that Nivolumab significantly improved OS, with a 1-year survival rate of 51%, compared to 39% in the Docetaxel group. Additionally, Nivolumab showed superior efficacy across all PD-L1 expression levels, with fewer grade 3–4 treatment-related adverse events (10% vs. 54%).

In the KEYNOTE-024 trial (2016), Pembrolizumab was investigated as a first-line treatment for advanced NSCLC in 305 patients with PD-L1 expression ≥ 50% [74]. Pembrolizumab significantly improved both overall and PFS compared to chemotherapy, with a 6-month survival rate of 80.2% in the Pembrolizumab group versus 72.4% in the chemotherapy group. Pembrolizumab also had a more favorable safety profile, with fewer serious adverse events.

The CheckMate 141 trial (2016) examined Nivolumab in patients with recurrent/metastatic squamous cell carcinoma of the head and neck (HNSCC) [75]. Nivolumab extended median OS to 7.5 months, compared to 5.1 months with standard therapy. Additionally, Nivolumab led to a 19 percentage-point higher 1-year survival rate and had fewer grade 3–4 treatment-related adverse events (13.1% vs. 35.1%), with stable quality of life in terms of physical, role, and social functioning.

The IMpassion130 trial (2018) assessed Atezolizumab combined with Nab-paclitaxel in patients with advanced TNBC. Atezolizumab plus Nab-paclitaxel demonstrated a median OS of 21.3 months, compared to 17.6 months with the placebo group [76]. The combination also improved progression-free survival (PFS), with a median PFS of 7.2 months in the intention-to-treat population, compared to 5.5 months with placebo. In the PD-L1-positive subgroup, PFS was 7.5 months vs. 5.0 months. No new adverse effects were identified; however, adverse events leading to discontinuation occurred in 15.9% of the Atezolizumab + Nab-paclitaxel group and 8.2% of the placebo group.

The OAK trial (2017) investigated Atezolizumab in previously treated NSCLC patients, with 1225 participants. Atezolizumab significantly improved OS, reaching a median of 13.8 months compared to 9.6 months with Docetaxel [77]. The study confirmed Atezolizumab’s potential as a treatment option for NSCLC with a favorable safety profile.

In the CheckMate 066 trial (2015), Nivolumab demonstrated improved overall survival compared to Dacarbazine in previously untreated advanced melanoma patients. The 1-year survival rates were 72.9% versus 42.1%, respectively [78]. Subgroup analyses from this study suggested the survival benefit was maintained regardless of BRAF mutation status, though the trial design did not specifically stratify patients by BRAF mutation.

The Avelumab trial (2016) for chemotherapy-refractory metastatic MCC involved 88 patients and demonstrated an ORR of 31.8%, with durable responses and minimal adverse effects [10]. This highlighted Avelumab as a promising new therapeutic option for this rare and aggressive cancer.

The Pembrolizumab versus Docetaxel trial (2015) involving 1034 patients with advanced NSCLC found that Pembrolizumab significantly improved OS and PFS compared to Docetaxel, particularly in patients with high PD-L1 expression, with fewer grade 3–5 adverse events [79].

These trials underscore the growing importance of ICIs in the treatment of various cancers. The use of ICIs has consistently shown substantial improvements in OS and PFS, with more favorable safety profiles when compared to conventional therapies. The results from these studies suggest that ICIs are becoming a pivotal part of the therapeutic landscape for advanced cancers, offering hope for patients who previously had limited treatment options. Moreover, ongoing research continues to explore the efficacy of ICIs across different cancer subtypes and treatment settings, emphasizing their potential as a cornerstone in cancer treatment. The promising outcomes observed so far highlight the significant role of immune checkpoint inhibition in reshaping cancer therapy, though further studies are necessary to fully understand the optimal use of these agents in diverse patient populations and clinical contexts. Some research cohorts that include multi-omics data have provided particularly valuable material for in-depth studies on the mechanisms of tumor immunotherapy. In a study examining two cohorts of melanoma patients, the researchers obtained baseline transcriptomic information from two cohorts of melanoma patients who received PD-1-based immunotherapy [80]. They used PFS to distinguish patients who benefited from the treatment from those who did not. The study demonstrated that baseline gene expression profiles could consistently predict long-term outcomes of immunotherapy with high precision. Another study has indicated that abnormal changes in DNA methylation are closely associated with the occurrence and development of cancer [81]. By analyzing various tumor samples, it was found that the loss of DNA methylation is significantly correlated with high mutation rates and increased copy number alterations in tumors. This association suggests that the loss of DNA methylation might be a mechanism by which tumor cells evade immune system surveillance. Further research has revealed that the loss of DNA methylation enhances the immunosuppressive capabilities of tumor cells by regulating the expression of specific genes, thereby promoting immune evasion of tumors.

In conclusion, cohorts, especially those with multi-omics data involved, have significantly advanced our understanding of the tumor immune microenvironment and the efficacy of ICIs. By leveraging these multi-dimensional approaches, researchers can better predict patient responses to treatment, uncover new therapeutic avenues, and ultimately improve clinical outcomes in cancer immunotherapy.

### 2.3. Prediction Models for Response to Tumor Immunotherapy

**Figure 2:**
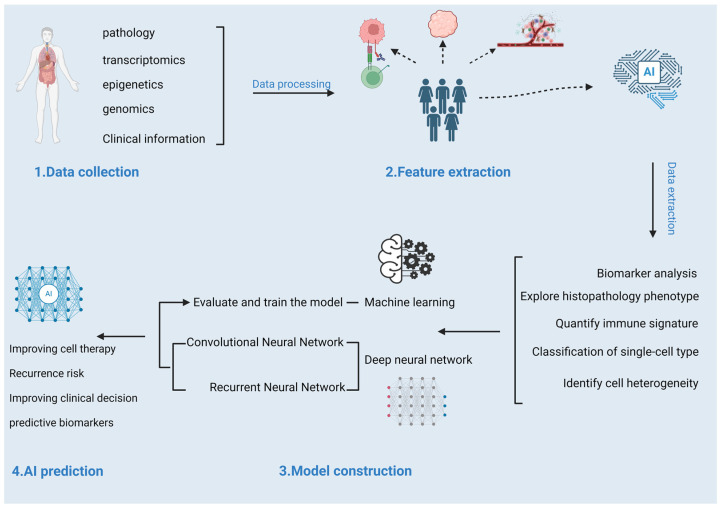
Integrating multi-omics data for AI-driven tumor immunotherapy: from data collection to predictive biomarkers.

Given the heterogeneity in patient responses to ICIs and the pivotal role of the TME in treatment efficacy, the integration of ML and deep learning models with multi-omics data and clinical parameters is increasingly recognized as a critical approach to enhance the predictive accuracy of ICI response in cancer immunotherapy. Against this background, the application of AI becomes particularly important. These advanced data analysis technologies can process large amounts of clinical data, genomic information, and other biomarkers to identify key factors affecting ICI efficacy [82]. They can also combine multi-omics and clinical data to demonstrate predictive value in immunotherapy and targeted therapy, improving accurate treatment effects for tumor patients [83]. The process is shown in Figure 2. To date, AI technology has shown extensive potential in the field of oncology, from tumor detection to prognosis prediction. In this section, we will explore the application of ML and deep neural networks (DNNs) in predicting responses to tumor immunotherapy.

#### 2.3.1. Machine Learning-Based Prediction Models

ML has revolutionized the field of oncology by enabling the development of predictive models that assist in early diagnosis, treatment stratification, and prognosis estimation for various cancer types. These models leverage complex algorithms to analyze large datasets, identifying patterns and correlations that may not be apparent through traditional statistical methods [84]. Among these features, PD-L1 expression levels, TMB, and MSI have been extensively studied [85]. Some models combine these biomarkers and use traditional machine learning algorithms for predictions across a single cancer type to multiple cancer types.

Recent studies highlight the growing role of machine learning (ML) in predicting immunotherapy responses through multi-omics integration. For example, an XGBoost-based model leveraging multi-omics data from TCGA demonstrated strong performance in cancer staging prediction (AUC: 0.595–0.872), suggesting its potential to stratify patients for immune checkpoint inhibitor (ICI) therapy [86]. In pan-cancer analyses, SVM and random forest models achieved robust classification accuracy (AUC: 0.742–0.787) across diverse malignancies, underscoring their utility in identifying biomarkers predictive of ICI efficacy [87,88]. Advanced frameworks like OncoNPC further exemplify this trend; trained on multi-omics data from 36,445 tumor patients, it improved genome-guided therapy selection and survival outcomes in cancer of unknown primary (weighted F1 score: 0.784) [89]. These models, integrating genomic, transcriptomic, and clinical features, pave the way for precision immunotherapy by linking complex omics patterns to treatment response.

#### 2.3.2. Deep Neural Network-Based Prediction Models

In addition to traditional ML algorithms, deep learning technology is also widely used in predicting responses to tumor immunotherapy. Deep learning constructs multi-layer neural networks that can automatically learn and extract complex features from data, thereby providing more precise prediction results. Convolutional neural networks (CNNs) stand out in predicting the effectiveness of ICIs by extracting complex features from medical images to achieve high-accuracy predictions. A study used a biologically guided deep learning method to train a multitask model that simultaneously predicted TME status and treatment outcomes from radiological images, and validated a model for predicting gastric cancer prognosis and the benefits of adjuvant chemotherapy in a multicenter international study. The results indicated that the model demonstrated a high level of discrimination, accurately classifying the tumor immune microenvironment and stromal microenvironment, with AUCs of 0.94–0.96 for internal and external validation cohorts, respectively [90]. Another study utilized deep learning to analyze PET/CT images for the non-invasive prediction of PD-L1 status and ICI treatment responses in non-small cell lung cancer. The study collected 18F-FDG-PET/CT images and clinical data from NSCLC patients and used a small residual convolutional network (SResCNN) to analyze these data, developing a deep learning score (DLS) to predict PD-L1 expression status. The results show that DLS can distinguish PD-L1 positive and negative expression, with AUCs of 0.89 and 0.84 in the training cohort. It was ultimately determined that it could serve as a prognostic biomarker guiding immunotherapy [91]. Recurrent neural networks (RNNs) are adept at handling time series data, capable of capturing time-dependent relationships during patient treatment [92]. The combination of RNN and CNN models also contributes to improving the accuracy of tumor prediction in radiology. For example, a study used transfer learning combined with CNN and RNN to analyze time series CT images of patients with locally advanced NSCLC to evaluate the effectiveness of deep learning networks in predicting clinical outcomes. The results showed that deep learning models using time series scans have significant predictive ability in predicting survival and cancer-specific outcomes, and with each additional follow-up scan, the model performance was enhanced, with an AUC of 0.74 [93]. With the advancement of deep learning technology, models based on complex neural network structures are also evolving. For example, a study proposed and validated a mutation-based deep learning model for survival analysis of patients receiving ICI therapy [94]. The interpretability of this model stems from biological perception, offering insights into novel genetic biomarkers. The results found that compared to the gold standard Cox-PH model (0.52 ± 0.10), the model had an average concordance index of 0.59 ± 0.13 across nine types of cancer, thereby producing impacts beyond ICI therapy. Overall, deep learning technology provides new tools and methods for predicting responses to tumor immunotherapy and plays a significant role in understanding the biological characteristics of tumors.

However, despite achieving remarkable results in many tasks, deep learning still faces some challenges in practical applications, such as data quality and quantity, model interpretability, and how to integrate deep learning models into clinical practice. Therefore, future research needs to further explore and address these issues to promote the application and development of deep learning in the field of tumor immunotherapy.

## 3. Discussion

In this review, we have explored the transformative potential of multi-omics data in predicting responses to ICI therapy, a critical advancement in precision oncology. By synthesizing insights from genomics, transcriptomics, proteomics, and epigenomics, we have outlined how these diverse layers of data contribute to a more comprehensive understanding of tumor biology and the TME, thus improving the accuracy of response predictions. The integration of these multi-omics features into predictive models marks a paradigm shift in how we approach cancer treatment, enabling personalized therapeutic strategies tailored to individual patient profiles.

ICIs, such as PD-1/PD-L1 and CTLA-4 inhibitors, have revolutionized cancer therapy. However, the response to these therapies is highly variable among patients, underscoring the need for robust predictive models to identify those most likely to benefit. Predicting ICI response not only maximizes therapeutic efficacy but also minimizes unnecessary side effects, making it a cornerstone of personalized treatment. The development of these predictive models has progressed from single biomarkers like TMB and MSI to more complex and nuanced multi-dimensional signatures. These advances underscore the increasing sophistication of predictive tools and the growing role of AI, particularly deep learning techniques, in improving predictive accuracy.

One of the most promising trends in ICI response prediction is the integration of multi-omics data with traditional biomarkers. Studies have demonstrated that the combination of genomic and transcriptomic data, along with epigenomic and proteomic features, yields a more comprehensive and accurate prediction of patient responses. Our own work, which combines DNA methylation profiles with gene expression data, highlights the potential of multi-layered omics approaches to improve model performance. These integrated models are poised to outpace traditional, single-layer biomarkers in terms of predictive power, offering a more nuanced view of the complex tumor–immune interactions that govern ICI efficacy.

Additionally, the application of DNNs in ICI response prediction has shown significant promise. While classical ML models, such as RFs and support vector machines, have been effective, DNNs can automatically extract complex patterns from large datasets, achieving superior predictive performance. This shift toward deep learning represents a major advancement in the field, as these models can handle high-dimensional data more effectively, offering the potential for improved accuracy and generalizability. Recent studies indicate that DNN-based models are increasingly becoming the standard for ICI response prediction, outstripping the performance of traditional methods.

Another exciting development is the move towards pan-cancer models. Traditionally, predictive models were developed for individual cancer types, but as more multi-omics data become available across different cancers, researchers are now building models that can predict ICI responses across a wide range of malignancies. This expansion is poised to increase the applicability of predictive tools, making them more versatile and widely applicable in clinical practice.

Despite these promising advances, significant challenges remain. A primary limitation is the lack of large, diverse clinical datasets, with many existing repositories, such as TCGA, falling short in terms of comprehensive clinical annotations related to ICI treatment. Moreover, the standardization and integration of data remain key issues, as data from different sources vary significantly in format, quality control, and preprocessing, which further hinders model development, integration, and validation. The absence of such data hinders the validation and clinical translation of predictive models, slowing progress toward real-world applications. Consequently, the integration of high-dimensional multi-omics data remains a computational challenge, necessitating the development of more sophisticated algorithms capable of handling the complexity of these datasets.

The mechanistic understanding of ICI resistance also remains underdeveloped. While predictive models based on multi-omics data have demonstrated impressive accuracy, understanding the underlying biological mechanisms of ICI resistance is essential for ensuring the long-term utility and interpretability of these models in clinical settings. Efforts to elucidate the genetic and epigenetic factors that drive resistance to immunotherapy will be crucial in refining these models and improving their clinical utility.

Ethical considerations are also an ongoing concern in the application of predictive models for ICI therapy. As multi-omics data collection becomes increasingly ubiquitous, issues related to patient privacy, data security, and the ethical use of predictive tools in clinical decision-making will need to be addressed. Ensuring that predictive models are used responsibly and transparently, with appropriate safeguards in place, will be essential for their successful integration into clinical practice.

In conclusion, the integration of multi-omics data into predictive models for ICI therapy is advancing rapidly, offering a more personalized approach to cancer treatment. These developments, particularly the use of deep learning and the shift toward pan-cancer models, represent a significant leap forward in oncology. However, challenges such as the need for large clinical datasets, the integration of complex data layers, and the ethical implications of using sensitive patient data must be navigated carefully. Future progress in this field will require continued collaboration among researchers, clinicians, and data scientists, as well as the development of more robust algorithms and ethical frameworks to ensure the safe and effective use of predictive models in cancer care.

In this review, we have explored the transformative potential of multi-omics data in predicting responses to ICI therapy, a critical advancement in precision oncology. By synthesizing insights from genomics, transcriptomics, proteomics, and epigenomics, we have outlined how these diverse layers of data contribute to a more comprehensive understanding of tumor biology and the TME. This, in turn, improves the accuracy of response predictions. The integration of these multi-omics features into predictive models marks a paradigm shift in how we approach cancer treatment, enabling personalized therapeutic strategies tailored to individual patient profiles.

ICIs, such as PD-1/PD-L1 and CTLA-4 inhibitors, have revolutionized cancer therapy. However, their variable response among patients underscores the need for robust predictive models to maximize therapeutic efficacy and minimize side effects. The development of these models has evolved from single biomarkers like TMB and MSI to complex multi-dimensional signatures. Deep learning techniques are playing an increasingly pivotal role in handling high-dimensional data and improving predictive accuracy. However, challenges remain. These include the lack of large, diverse clinical datasets with comprehensive ICI treatment annotations, the standardization and integration of multi-omics data, and the interpretability of complex models, particularly DNNs. As predictive models become more complex, especially with the incorporation of deep learning algorithms, understanding how they arrive at specific predictions becomes increasingly difficult. Transparent and interpretable models are essential for clinical decision-making. Lack of interpretability can hinder the widespread adoption and integration of these advanced predictive tools into clinical practice.

Meanwhile, experimental models have emerged as valuable tools for validating predictive models of ICI response. These systems not only enable assessment of predictive accuracy but also facilitate a more tailored approach to therapy selection. They ensure that patients receive optimal treatments based on tumor characteristics. Additionally, emerging modalities such as tumor organoids—which replicate the tumor microenvironment with greater fidelity than traditional cell lines—offer an alternative platform for validating ICI activity predictions and conducting personalized drug screening. Ethical considerations are also an ongoing concern in the application of predictive models for ICI therapy. As multi-omics data collection becomes increasingly ubiquitous, issues related to patient privacy, data security, and the ethical use of predictive tools in clinical decision-making will need to be addressed. Ensuring that predictive models are used responsibly and transparently, with appropriate safeguards in place, will be essential for their successful integration into clinical practice.

In conclusion, the integration of multi-omics data into predictive models for ICI therapy is advancing rapidly, offering a more personalized approach to cancer treatment. These developments, particularly the use of deep learning and the shift toward pan-cancer models, represent a significant leap forward in oncology. However, challenges such as the need for large clinical datasets, the integration of complex data layers, and the ethical implications of using sensitive patient data must be navigated carefully. Future progress in this field will require continued collaboration among researchers, clinicians, and data scientists, as well as the development of more robust algorithms and ethical frameworks, to ensure the safe and effective use of predictive models in cancer care.

## 4. Conclusions

ICI response prediction is crucial for advancing immunotherapy. Recent developments in high-throughput sequencing and bioinformatics have led to the identification of key biomarkers and predictive algorithms, with significant research data accumulated. The field is moving toward integrating multi-dimensional data and utilizing advanced AI algorithms to enhance predictive models. These advances hold great potential for improving personalized cancer treatments.

## Figures and Tables

**Table 1:** ICI response evaluation studies—classic cohorts.

Study	Name	Published Year	Countries	Cancer Type	Number of Participants	Drugs Used	Treatment Duration	Survival	Key Findings
Snyder et al. (2015) [69]	CheckMate 057: Nivolumab in Non-Small-Cell Lung Cancer (NSCLC)	2015	Global	NSCLC	582	Nivolumab, Docetaxel	Median follow-up of 18 months	1-year: Nivolumab 51%, Docetaxel 39%; 18-month: Nivolumab 39%, Docetaxel 23%	Nivolumab significantly improved overall survival compared to Docetaxel in advanced non-squamous NSCLC after platinum-based chemotherapy. Nivolumab showed superior efficacy across all PD-L1 expression levels (≥1%, ≥5%, ≥10%). Lower incidence of grade 3–4 treatment-related adverse events in Nivolumab group (10% vs. 54%).
Reck et al. (2016) [74]	KEYNOTE-024: Pembrolizumab for NSCLC (First-Line Therapy)	2016	Global	NSCLC	305	Pembrolizumab, platinum-based chemotherapy	Median follow-up not specified, progression-free survival assessed	6-month overall survival: Pembrolizumab 80.2%, Chemotherapy 72.4%	Pembrolizumab significantly improved progression-free and overall survival compared to chemotherapy in previously untreated advanced NSCLC with PD-L1 ≥50% expression and no EGFR or ALK mutations. Fewer grade 3–5 treatment-related adverse events in the Pembrolizumab group (26.6% vs. 53.3%).
Ferris et al. (2016) [75]	CheckMate 141: Nivolumab in Head and Neck Squamous Cell Carcinoma (HNSCC)	2016	Global (North America, Europe, Asia)	HNSCC	361	Nivolumab (anti-PD-1 monoclonal antibody)	Median follow-up not specified, progression-free survival assessed	Median overall survival: Nivolumab 7.5 months, standard therapy 5.1 months; 1-year survival: Nivolumab 36.0%, standard therapy 16.6%	Nivolumab significantly prolonged overall survival compared to standard therapy (hazard ratio 0.70, *p* = 0.01). The 1-year survival rate for Nivolumab was 19 percentage points higher. Median progression-free survival was 2.0 months for Nivolumab and 2.3 months for standard therapy. Nivolumab had a lower incidence of grade 3–4 treatment-related adverse events (13.1% vs. 35.1%) and maintained stable physical, role, and social functioning, while these worsened with standard therapy.
Emens et al. (2018) [76]	IMpassion130: Atezolizumab in Triple-Negative Breast Cancer (TNBC)	2018	International multicenter study	TNBC	902	Atezolizumab (anti-PD-L1 monoclonal antibody), Nab-paclitaxel	Median follow-up of 12.9 months	Median overall survival: Atezolizumab + Nab-paclitaxel 21.3 months, Placebo + Nab-paclitaxel 17.6 months	In the intention-to-treat population, median progression-free survival was 7.2 months for Atezolizumab + Nab-paclitaxel, compared to 5.5 months for placebo + Nab-paclitaxel. In the PD-L1-positive subgroup, progression-free survival was 7.5 months vs. 5.0 months. No new adverse effects were identified; adverse events leading to discontinuation occurred in 15.9% of the Atezolizumab group and 8.2% of the placebo group.
Rittmeyer et al. (2017) [77]	OAK Trial: Atezolizumab in NSCLC (Second-Line Therapy)	2017	31 countries	NSCLC	1225	Atezolizumab, Docetaxel	Median follow-up of 12.6 months	Median overall survival (ITT): Atezolizumab 13.8 months, Docetaxel 9.6 months	Atezolizumab significantly improved overall survival compared to Docetaxel in previously treated NSCLC patients, with a favorable safety profile.
Robert et al. (2015) [78]	CheckMate 066: Nivolumab in Advanced Melanoma	2015	Not specified	Melanoma	418	Nivolumab, Dacarbazineprovided	Specific follow-up time not provided	1-year overall survival: Nivolumab 72.9%, Dacarbazine 42.1%	Nivolumab significantly improved overall survival and progression-free survival compared to Dacarbazine in previously untreated patients with advanced melanoma without BRAF mutation.
Kaufman et al. (2016) [10]	Avelumab in Patients with Chemotherapy-Refractory Metastatic Merkel Cell Carcinoma	2016	North America, Europe, Australia, and Asia	MCC	88	Avelumab (Bavencio)	Median follow-up of 10.4 months	Objective response rate: 31.8% (28 of 88 patients, 95% CI 21.9–43.1)	Avelumab was associated with durable responses, most of which are still ongoing, and was well tolerated. Avelumab represents a new therapeutic option for advanced Merkel cell carcinoma.
Herbst et al. (2015) [79]	KEYNOTE-010: Pembrolizumab in Advanced NSCLC	2015	24 countries	NSCLC	1034	Pembrolizumab, Docetaxel	Median overall survival: Pembrolizumab 2 mg/kg: 10.4 months, Pembrolizumab 10 mg/kg: 12.7 months, Docetaxel: 8.5 months	Overall survival significantly longer for Pembrolizumab 2 mg/kg (HR 0.71, 95% CI 0.58–0.88, *p* = 0.0008) and Pembrolizumab 10 mg/kg (HR 0.61, 95% CI 0.49–0.75, *p* < 0.0001) compared to Docetaxel	In PD-L1 ≥50% population, overall survival was significantly longer with Pembrolizumab (2 mg/kg: 14.9 months vs. Docetaxel 8.2 months, HR 0.54, *p* = 0.0002; 10 mg/kg: 17.3 months vs. Docetaxel 8.2 months, HR 0.50, *p* < 0.0001). Progression-free survival was also significantly longer with Pembrolizumab (2 mg/kg: 5.0 months vs. Docetaxel 4.1 months, HR 0.59, *p* = 0.0001; 10 mg/kg: 5.2 months vs. Docetaxel 4.1 months, HR 0.59, *p* < 0.0001). Grade 3–5 adverse events were less common with Pembrolizumab (13% for 2 mg/kg, 16% for 10 mg/kg) compared to Docetaxel (35%).

## Data Availability

No data were used for the research described in this article.

## Supplementary Materials

The following supporting information can be downloaded at: https://www.mdpi.com/article/10.3390/life15020283/s1, Supplementary Table S1: Medical Terms: Full Name and Explanation.
