# Supplementary material for: Towards the Prediction of Responses to Cancer Immunotherapy: A Multi-Omics Review
Source: Life (Basel). 2025 Feb 12;15(2):283. doi: 10.3390/life15020283 (PMC11856636; doi:10.3390/life15020283)
Supplement: Supplementary file 1 [file life-15-00283-s001.zip › life-3423151-supplementary.pdf]

Supplementary Table S1. Medical Terms: Full Name and Explanation.

Abbreviation	Full Term	Explain
AI	Artificial Intelligence	AI refers to the simulation of human intelligence in machines programmed to think and learn like humans.It is used in various fields including healthcare for tasks such as diagnosis and personalized treatment recommendations.
TMB	Tumor Mutation Burden	TMB is a measure of the total number of mutations within a tumor's genome.<br>Higher TMB is often associated with better responses to immunotherapy.
ICI	Immune Checkpoint Inhibitor	ICIs are drugs that help the immune system recognize and attack cancer cells.<br>Examples include pembrolizumab and nivolumab, which block proteins like PD-1/PD-L1.
NSCLC	Non-Small Cell Lung Cancer	NSCLC is the most common type of lung cancer, accounting for about 85% of cases.<br>It includes subtypes such as adenocarcinoma and squamous cell carcinoma.
MCC	Merkel Cell Carcinoma	MCC is a rare and aggressive skin cancer that typically appears on or near the skin.<br>It is often associated with Merkel cell polyomavirus (MCV) infection.
CRC	Colorectal Cancer	CRC refers to cancer that starts in the colon or rectum.<br>Early screening can significantly improve outcomes through removal of polyps before they become

		cancerous.
PFS	Progression-Free Survival	PFS is the length of time during and after treatment when a patient's disease does not get worse.<br>It is an important metric in oncology clinical trials.
ORR	Objective Response Rate	ORR measures the percentage of patients who experience a significant reduction in tumor size.<br>It is used to evaluate the effectiveness of cancer treatments.
MSI	Microsatellite Instability	MSI is a condition where there are errors in the DNA replication process, leading to instability in certain areas of the genome.<br>It is commonly seen in some types of colorectal and endometrial cancers.
dMMR	Deficient Mismatch Repair	dMMR indicates a defect in the mismatch repair genes, which normally fix mistakes during DNA replication.<br>Deficiencies can lead to increased mutation rates and are linked to certain cancers.
GBC	Gallbladder Cancer	GBC is a rare but aggressive cancer that originates in the gallbladder, the organ that stores bile.<br>Surgery is the primary treatment if diagnosed early.
PSCC	Penile Squamous Cell Carcinoma	PSCC is a type of cancer affecting the penis, usually caused by human papillomavirus (HPV) infection.<br>Treatment options include surgery, radiation, and chemotherapy.
PD-L1	Programmed Death-Ligand 1	PD-L1 is a protein expressed

		on the surface of tumor cells that helps them evade detection by the immune system.<br>Inhibiting PD-L1 can enhance the body’s immune response against cancer cells.
NAL	Neoantigen Load	NAL refers to the number of neoantigens (new antigens formed from mutated proteins) presented by a tumor.<br>Higher neoantigen load can potentially make tumors more visible to the immune system.
ITS	ImmunoTherapy Score	ITS estimates a patient's likelihood of responding to immunotherapy based on the tumor's genetic and immunological characteristics.<br>It helps in predicting which patients might benefit most from immunotherapy treatments.
USH2A	Usher Syndrome 2A	USH2A is a gene associated with Usher syndrome, a genetic disorder causing hearing loss and progressive blindness.<br>Mutations in this gene can lead to syndromic hearing impairment and retinitis pigmentosa.
ZFHX4	Zinc Finger Homeobox 4	ZFHX4 is a transcription factor involved in regulating gene expression.<br>Dysregulation of ZFHX4 has been implicated in certain types of cancer.
PLCO	Prostate, Lung, Colorectal, and Ovarian	PLCO stands for a group of cancer types that includes prostate, lung, colorectal, and ovarian cancers.<br>These cancers are among the most common and extensively

		studied malignancies worldwide.
GBM	Glioblastoma	GBM is a highly aggressive brain tumor with poor prognosis.<br>It is characterized by rapid growth and invasion into surrounding brain tissue.
TME	Tumor MicroEnvironment	TME comprises the cellular environment surrounding a tumor, including immune cells, fibroblasts, and blood vessels.<br>The composition of TME can influence tumor progression and response to therapy.
MMR	Mismatch Repair	MMR is a group of proteins responsible for fixing errors that occur during DNA replication.<br>Defects in MMR can lead to genetic instability and an increased risk of certain cancers.
HRD	Homologous Recombination Deficiency	HRD refers to a defect in the cell's ability to repair double-strand breaks in DNA.<br>Tumors with HRD are more likely to respond to certain types of cancer therapy, like PARP inhibitors.
APOBEC	Apolipoprotein B mRNA Editing Enzyme Catalytic Subunit	APOBEC enzymes edit RNA molecules and can induce mutations in DNA.<br>They play a role in the immune response against viruses but can also contribute to cancer development.
WGS	Whole-Genome Sequencing	WGS involves sequencing the entire genome of an organism to identify genetic variations and mutations.<br>It provides comprehensive information about genetic alterations in cancer.

PCAWG	Pan-Cancer Analysis of Whole Genomes	PCAWG aims to analyze genomic data from multiple cancer types to understand common patterns and differences.<br>It helps identify genetic drivers of cancer and potential therapeutic targets across different cancers.
IFN-γ	Interferon-Gamma	IFN-γ is a cytokine produced by immune cells that plays a crucial role in fighting viral infections and regulating immune responses.<br>It can also have antitumor effects by promoting immune cell activity against cancer cells.
scRNA-seq	Single-Cell RNA Sequencing	scRNA-seq allows for the analysis of gene expression at the single-cell level.<br>It provides insights into the heterogeneity of cell types within a tumor and their functions.
TILs	Tumor-Infiltrating Lymphocytes	TILs are immune cells that have migrated into the tumor and can indicate a robust antitumor immune response.<br>Their presence is often associated with better clinical outcomes in some cancers.
Tregs	Regulatory T Cells	Tregs are a subset of T cells that suppress immune responses to maintain tolerance to self-antigens.<br>In cancer, high levels of Tregs can impair the immune system's ability to fight tumor cells.
MDSCs	Myeloid-Derived Suppressor Cells	MDSCs are a diverse group of cells that suppress T cell responses and promote tumor progression.

		Targeting MDSCs is a strategy to enhance antitumor immunity.
MHC	Major Histocompatibility Complex	A set of cell surface proteins essential for immune recognition,presenting antigens to T cells to initiate immune responses.
H3K27me3	Histone H3 Lysine 27 Trimethylation	A repressive epigenetic mark on histone H3, associated with gene silencing and heterochromatin formation.
H3K4me3	Histone H3 Lysine 4 Trimethylation	An activating epigenetic mark on histone H3, enriched at promoter regions of transcriptionally active genes.
H3K56ac	Histone H3 Lysine 56 Acetylation	An epigenetic modification involved in DNA replication and repair by loosening chromatin structure.
H4K20me2	Histone H4 Lysine 20 Dimethylation	A histone modification linked to chromatin compaction, DNA repair, and transcriptional regulation.
H4K16ac	Histone H4 Lysine 16 Acetylation	An epigenetic mark associated with open chromatin, promoting transcriptional activation and DNA accessibility.
HDAC6	Histone Deacetylase 6	HDAC enzymes remove acetyl groups from histone proteins, tightening chromatin structure and reducing gene expression. HDAC inhibitors are used in cancer therapy to reactivate silenced genes involved in tumor suppression.
KMT2A	Lysine Methyltransferase 2A	KMT2A is a histone lysine methyltransferase responsible for catalyzing H3K4 methylation, playing a crucial role in gene regulation and development. It is involved in

		various biological processes, including hematopoiesis and cellular development, and its alterations are associated with multiple diseases.
BRD4	Bromodomain Containing 4	BRD4 is an epigenetic regulator that controls gene expression by binding to acetylated histones and recruiting transcriptional machinery. It is implicated in cancer progression and cell proliferation, and its inhibition can induce cell death through various pathways, making it a promising therapeutic target.
ML	Machine Learning	ML is a subset of AI that uses algorithms to learn patterns from data and make predictions or decisions without being explicitly programmed.<br>In healthcare, ML is used for tasks such as medical imaging analysis, predictive diagnostics, and personalized treatment recommendations.
DNN	Deep Neural Network	DNNs are a class of artificial neural networks with many hidden layers, capable of learning complex patterns in large datasets.<br>They are used in image recognition, natural language processing, and other advanced computational tasks.
SVM	Support Vector Machine	SVM is a supervised learning algorithm used for classification and regression tasks in machine learning. It aims to find the optimal hyperplane that maximizes the margin between different

		classes, making it effective for complex data analysis and predictive modeling.
RF	Random Forest	Random Forest is an ensemble learning method that constructs multiple decision trees to improve predictive accuracy and robustness. It is widely used for classification and regression tasks, offering high performance and the ability to handle large datasets with minimal overfitting.
RFS	Recurrence-Free Survival	RFS measures the length of time after treatment during which there is no sign of recurrent disease.<br>It is an important endpoint in cancer clinical trials assessing adjuvant therapies.
AUC	Area Under the Curve	
RCI	Rank Correlation Index	RCI is a statistical measure used to evaluate the strength and direction of association between two ranked variables.<br>It is particularly useful in analyzing ordinal data or non-parametric statistics.<br>In the context of machine learning models, RCI might be used to assess the relationship between predicted outcomes and actual results, providing insights into model performance and consistency.
CNN	Convolutional Neural Network	CNN is a deep learning model commonly used for image recognition and processing. It uses convolutional layers to extract features from input data, making it highly effective for tasks like image classification and object detection.

RNN	Recurrent Neural Network	RNN is a type of neural network designed to handle sequential data by maintaining a hidden state that captures information from previous inputs. It is widely used in natural language processing and time-series analysis.
SResCNN	Small Residual Convolutional Network	SResCNN is a variant of convolutional neural networks that incorporates residual connections to improve training efficiency and performance. It is particularly useful for tasks requiring high accuracy with limited computational resources.
DLS	Deep Learning Score	DLS is a metric or scoring system used to evaluate the performance of deep learning models. It often combines multiple factors such as accuracy, precision, and recall to provide a comprehensive assessment of model effectiveness.
CUP	Cancer of Unknown Primary	CUP refers to a type of cancer where the original site of the tumor is not identified at the time of diagnosis. It is a challenging clinical scenario that often requires extensive testing and advanced diagnostic techniques to determine the best treatment approach.
XGBoost	Extreme Gradient Boosting	XGBoost is an advanced machine learning algorithm that uses gradient boosting to create an ensemble of weak prediction models, typically decision trees. It is highly efficient and widely used for classification and regression

		tasks due to its strong predictive performance and scalability.
TCGA	The Cancer Genome Atlas	TCGA is a comprehensive and coordinated effort to accelerate the understanding of the molecular basis of cancer through the application of genome analysis technologies, including large-scale genome sequencing. It provides valuable data for cancer research and the development of targeted therapies.
5 mC	5-Methylcytosine	5-Methylcytosine (5mC) is a chemical modification of cytosine bases in DNA, where a methyl group is added to the cytosine ring. It is an important epigenetic marker involved in gene regulation, genomic imprinting, and the suppression of transposable elements.
